# A novel role for decadienyl-L-carnitine in pulmonary vascular remodeling and the underlying interventional mechanism of osthole

**DOI:** 10.1186/s13020-026-01362-8

**Published:** 2026-03-16

**Authors:** Yuan Li, Dongyang Han, Junjie Liu, Yuxin Qiao, Jiaoxia Wei, Haitao Lu, Li Yao

**Affiliations:** 1 Department of Medicinal Chemistry and Natural Medicine Chemistry, Department of Pharmacognosy, College of Pharmacy, Harbin Medical University, Harbin, 150081 China; 2 State-Province Key Laboratory of Biomedicine-Pharmaceutics of China, Harbin Medical University, Harbin, 150081 China; 3 School of Chinese Medicine, Hong Kong Traditional Chinese Medicine Phenome Research Center, Hong Kong Baptist University, Hong Kong, 999077 China; 4 Key Laboratory of Systems Biomedicine (Ministry of Education), Shanghai Center for Systems Biomedicine, State Key Laboratory of Medical Genomics, Ruijin Hospital, Shanghai Jiao Tong University, Shanghai, 200240 China

**Keywords:** Osthole, Decadienyl-L-carnitine, NLRP3, Pyroptosis; pulmonary vascular remodeling

## Abstract

**Background:**

Pulmonary hypertension (PH) is a severe pulmonary vascular disease lacking early diagnostic biomarker and effective therapeutics. Osthole has capability to alleviate pulmonary vascular remodeling targeting by decadienyl-L-carnitin (C10:2) in PH rats. We sought to explore the novel functional mechanism of C10:2 in cell proliferation, apoptosis, extracellular matrix remodeling, and energy biosynthesis of pulmonary vascular remodeling as well as new inventional mechanism of osthole.

**Methods:**

Animal and cell models of PH were established using monocrotaline (MCT) and platelet-derived growth factor-BB (PDGF-BB). C10:2 biosynthesis was manipulated through the administration of exogenous C10:2 and etomoxir. Markers of pyroptosis and pulmonary vascular remodeling, as well as components of the C10:2/HSP47/NLRP3 axis, were evaluated using western blotting, ELISA, and biochemical assays.

**Results:**

Osthole inhibited cell pyroptosis and alleviated pulmonary vascular remodeling by suppressing the expression of NLRP3, GSDMD, Caspase-1, IL-1β, IL-18, and C10:2 in PH rats. Additionally, C10:2 levels were positively correlated with the progression of pulmonary vascular remodeling in a time-dependent manner. C10:2, similar to PDGF-BB, promoted the proliferation of pulmonary arterial smooth muscle cells (PASMCs), accelerated extracellular matrix remodeling, inhibited apoptosis, activated AMPKα-1, and increased ROS accumulation, ultimately leading to mitochondrial dysfunction in PASMCs. Osthole attenuated C10:2-induced pulmonary vascular remodeling by downregulating proliferation markers (PCNA, cyclin A, CDK2), modulating apoptosis markers (Caspase-3, Bax, Bcl-2), inhibiting migration-related proteins (MMP2, MMP9, TGF-β), and reducing AMPKα-1 and ROS overaccumulation as well as HSP47 expression. Collectively, our findings reveal a novel role for C10:2 in accelerating pulmonary vascular remodeling by promoting proliferation, apoptosis resistance, extracellular matrix remodeling, and mitochondrial dysfunction through NLRP3 inflammasome activation. Mechanistically, osthole significantly inhibited pyroptosis and mitigated pulmonary vascular remodeling via the C10:2/HSP47/NLRP3 axis.

**Conclusion:**

Our study identifies a novel function of C10:2 in promoting pyroptosis and accelerating pulmonary vascular remodeling through activation of the HSP47/NLRP3 axis. Furthermore, we demonstrate that osthole effectively inhibits C10:2/HSP47/NLRP3 axis-induced pyroptosis, thereby alleviating pulmonary vascular remodeling. These findings suggest that C10:2 may serve as a potential biomarker for PH diagnosis and provide a foundation for the development of novel anti-PH therapeutic strategies.

**Graphical Abstract:**

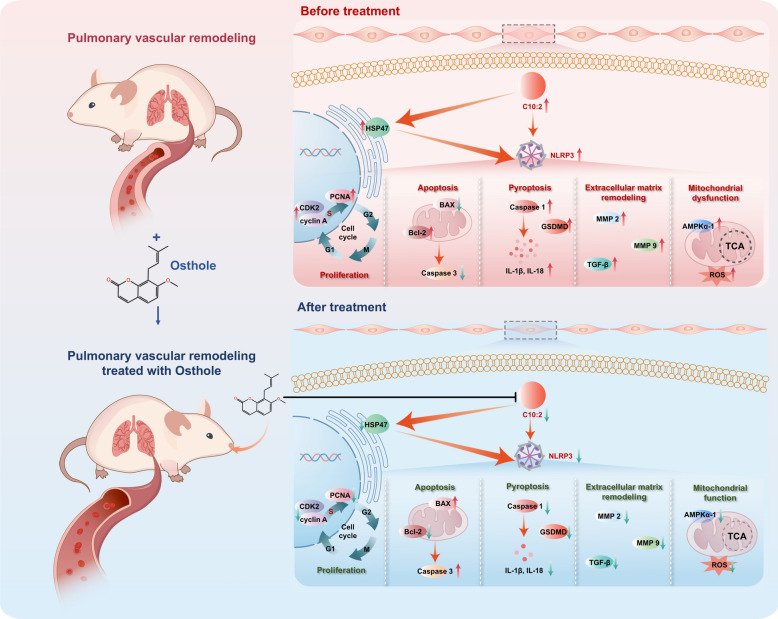

**Supplementary Information:**

The online version contains supplementary material available at 10.1186/s13020-026-01362-8.

## Introduction

Pulmonary hypertension (PH) is a deadly pulmonary vascular disease due to the lack of early diagnostic practice and effective interventions [1, 2]. Early and precise diagnosis of PH is clinical challenge and still relies on invasive examination- right heart catheterization [3]. A novel diagnostic biomarker is urgently needed allowing for non-invasive rapid screening, facilitating PH diagnosis and replying for therapy [4]. Pulmonary vascular remodeling(PVR) is the crucial pathological hallmark for increase of pulmonary arterial pressure, and critical for PH treatment[5]. Discovering early diagnostic biomarkers and developing effective drug targeting for PVR would be critical issue for PH to be addressed.

Osthole, a natural product derived from Chinese herbal medicine Cnidium monnieri (L.) Cusson and Angelica pubescens Maxim, is a promising candidate drug for the treatment of PH[6, 7]. Studies have shown osthole relaxes isolated pulmonary arteries of humans and rats by activating PI3K/AKT/eNOS/NO signaling pathway [8]. Osthole promotes PASMCs apoptosis by modifying Bax/Bcl-2-caspase 3 signal pathway and inhibits PASMCs proliferation by regulating TGF-β1/Smad/p38 signaling pathway [9, 10]. Notably, we found the protein targets of osthole majorly accounts for metabolic pathways [6]. Additionally, we delineate that osthole reduces serum sphingosine-1-phosphate (S1P) to reduce pulmonary arterial pressure [7], and inhibits decadienyl-l-carnitine(C10:2) overaccumulation to alleviate PVR in PH rats[5]. Therefore, manipulating C10:2 level by drugs appears to provide a new strategy to treat PVR in PH.

C10:2 is classified as a medium-chain acylcarnitine, whose primary biological function is to facilitate the bidirectional transport of acyl groups from the cytosol into the mitochondrial matrix for fatty acid β-oxidation. This process enables energy production necessary for cell viability and the maintenance of metabolic homeostasis [11, 12]. Medium-chain acylcarnitine, as an invaluable biomarker for inherited diseases correlated with fatty acid metabolic disorder, is detected in various body fluids (e.g.,plasma, serum or urine) [13]. Basically, serum medium-chain acylcarnitine(C6-C12) was reported to be increased in PH patients [14], and associated with key clinical symptoms 6-min-walk distances and N-terminal pro brain natriuretic peptide(NT-proBNP) [15]. NT-proBNP is considered an auxiliary screening biomarker for pulmonary hypertension (PH), as its levels correlate with disease severity and prognosis in patients; however, its specificity remains limited [16]. These studies demonstrates that medium-chain C10:2 likely represents emerging biomarker for PH [17]. Our previous work demonstrated that C10:2 promotes cell pyroptosis, thereby facilitating PVR in PH rats [18]. However, the causal relationship, downstream signaling pathways, and the crosstalk between C10:2-induced pyroptosis and other cellular phenotypes involved in PVR remain unclear.

To decipher functional mechanisms of C10:2 in PVR and delineate invention mechanism of osthole against PVR, we first analyzed the correlation between C10:2 and development process of PVR, then investigated the effect of osthole on cell pyroptosis. Furthermore, we detected the function of C10:2 in PASMCs proliferation, apoptosis, extracellular matrix remodeling, mitochondrial function, to delineate functional mechanism of C10:2. Finally, we explored downstream signaling pathway of C10:2 in PVR. Importantly, based on these new findings, we revealed the modulation mechanisms that osthole retards cell pyroptosis to ameliorate PVR by controlling C10:2 level.

## Materials and methods

### Chemicals and reagents

Osthole (purity 99%, Lot#: L2122261), Etomoxir (purity 98%, Lot#: L2525402), Decadienyl-L-carnitine (purity 98%, Lot#: I2503394), MCC950 (purity 97%, Lot#: J2530512) were procured from Aladdin Co. (Shanghai, China).

### Animals

Male Sprague–Dawley rats (weighing 200 ± 10 g) were obtained from the Experimental Animal Center of Harbin Medical University, which is fully accredited by the Institutional Animal Care and Use Committee (IACUC) of Harbin Medical University (Approval No.: IRB3091724). According to the previous study [5, 19], PH was induced in rats by intraperitoneal injection of MCT (60 mg·kg^−1^), while the control group received an equivalent volume of saline. Subsequently, drugs were orally administered for 28 days, including osthole (40, 80 mg·kg^−1^) and Sildenafil (35 mg·kg^−1^).

### HE

Paraffin sections are deparaffinized and hydrated, sequentially immersed in hematoxylin and eosin. Then, the sections are transferred into graded ethanol (70%, 80%, 95%, 100%) for gradual dehydration, followed by xylene to make the sections transparent. Neutral resin is used for mounting to avoid bubbles, the slides are baked overnight and then observed under a microscope.

### Cell culture

PASMCs(#HTX2067, OTWO) were incubated in DMEM medium containing 10% fetal bovine serum (FBS) at 37 °C in a 5% CO_2_ incubator for 24 h. According to experimental requirements, the cells exposed to PDGF-BB (40 ng·ml^−1^) to induce a classic cell model of PH, followed by administration of different doses of othole, etomoxir, and C10:2.

### MTT assay

PASMCs were seeded in 96-well plates, further treated with different concentrations of the agents. After culturing at 37 °C for 24 h, the cells were added MTT reagent (Biosharp, China) with continuous incubation for 4 h. Then DMSO were added in the dark and the absorbance was measured at 492 nm using a microplate reader.

### Western blotting

Protein samples were separated by electrophoresis and transferred to a nitrocellulose membrane (Millipore, USA), blocked with 5% non-fat milk for 2 h, and then incubated overnight at 4 °C with primary antibodies specific for PCNA (WANLEIBIO), cyclin A (WANLEIBIO), CDK2 (Bioss), TGF-β (Bioss), Caspase 3 (Bioss), Bax (Boster), Bcl-2 (Bioss), MMP2 (Bioss), MMP9 (Bioss), AMPKα−1 (Bioss), HSP47 (Boster), NLRP3 (WANLEIBIO), Caspase 1 (WANLEIBIO), IL-18 (Affinty), and IL-1β (Bioss). Then, they were incubated with secondary antibodies for 1 h. Western blotting was scanned using Image Studio software.

### ELISA

In ELISA kit (Meimian), samples and working solution are added sequentially, and the optical density (O.D) value is measured at 450 nm. The concentration of each sample is calculated using the standard curve linear regression equation.

### Examination of ROS

ROS was assessed with an Assay Kit from Beyotime (Shanghai, China). PASMCs were collected and suspended in diluted DCFH-DA (10 μmol·L^−1^) with a concentration of 1 million to 20 million·ml^−1^ and incubated at 37℃ for 20 min. Mix upside down every 3–5 min so that the probe is in full contact with the cell. Then PASMCs were washed three times to fully remove DCFH-DA that did not enter the cells. Fluorescence microscope was employed to take images (Nexcope, China), and Image J was performed to calculate average fluorescence intensity.

### Statistical analysis

Statistical analysis was performed with GraphPad Prism 9 software. The statistical significance was analyzed using one-way analysis of variance (ANOVA). Data were presented as mean ± S.E.M., statistical significance was set at *p* < 0.05.

## Results

### Osthole inhibited pyroptosis and C10:2 level to alleviate pulmonary vascular remodeling in PH rats

**Fig. 1:**
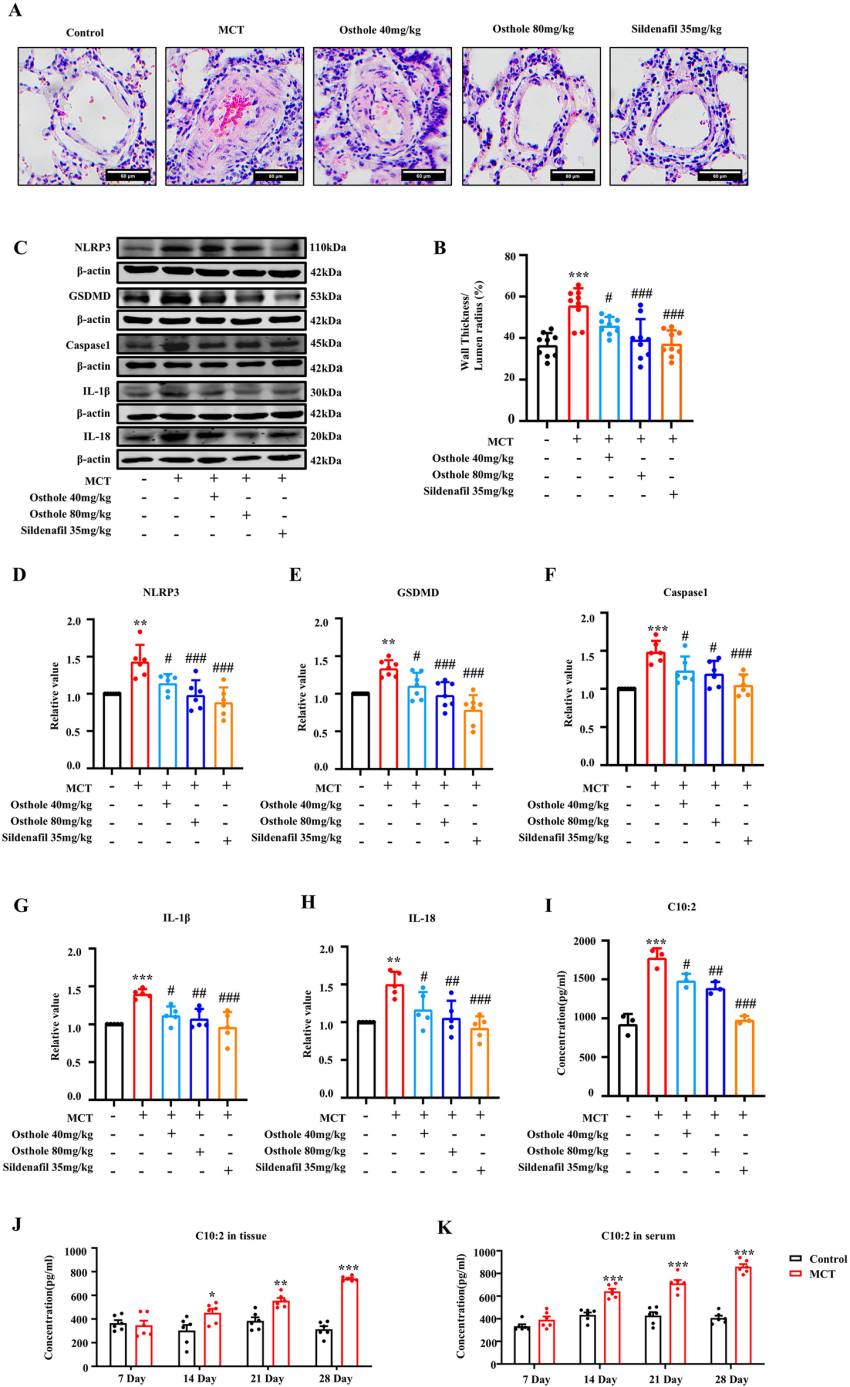
Osthole inhibited cell pyroptosis and reduced C10:2 levels, thereby alleviating pulmonary vascular remodeling in PH rats. **A** HE staining of lung tissue (n = 9). **B** Quantitative analysis of HE staining. **C**–**H** Expression levels of NLRP3, GSDMD, caspase-1, IL-1β, and IL-18 in lung tissue (n ≥ 5). **I**, **J** C10:2 levels in lung tissue (n = 6). **K** C10:2 levels in serum (n = 6). Data were represented as mean ± SEM. *p < 0.05, **p < 0.01, ***p < 0.001, compared with Control group; ^#^*p* < 0.05,^##^*p* < 0.01, ^###^*p* < 0.001, compared with MCT-induced PH group

To investigate the effect of osthole on pyroptosis and PVR, HE staining and pyroptosis markers of pulmonary vascular were evaluated in rats [20]. As expected, successful animal model of PH were established by MCT, and osthole could alleviate PVR by reducing wall thickness of pulmonary vascular in PH rats [5, 7] (Fig. 1A-B). Simultaneously, pyroptosis markers NLRP3, GSDMD, caspase1, IL-1β and IL-18were all significantly upregulated, indicating pyroptosis occurred in MCT induced PH rats (Fig. 1C-H), while these changes were all restored by osthole treatment. Moreover, C10:2 levels in lung tissue and serum were increased gradually along with time 7 d, 14 d, 21 d and 28 d at the development process of PVR in PH rats respectively (Fig. 1I-K). These findings suggested that C10:2 is positively correlated with PVR development, additionally, osthole obviously inhibited pyroptosis to alleviate PVR by reversing C10:2 level.

### C10:2 directly promoted PASMCs proliferation and reversed by osthole treatment in vitro

**Fig. 2:**
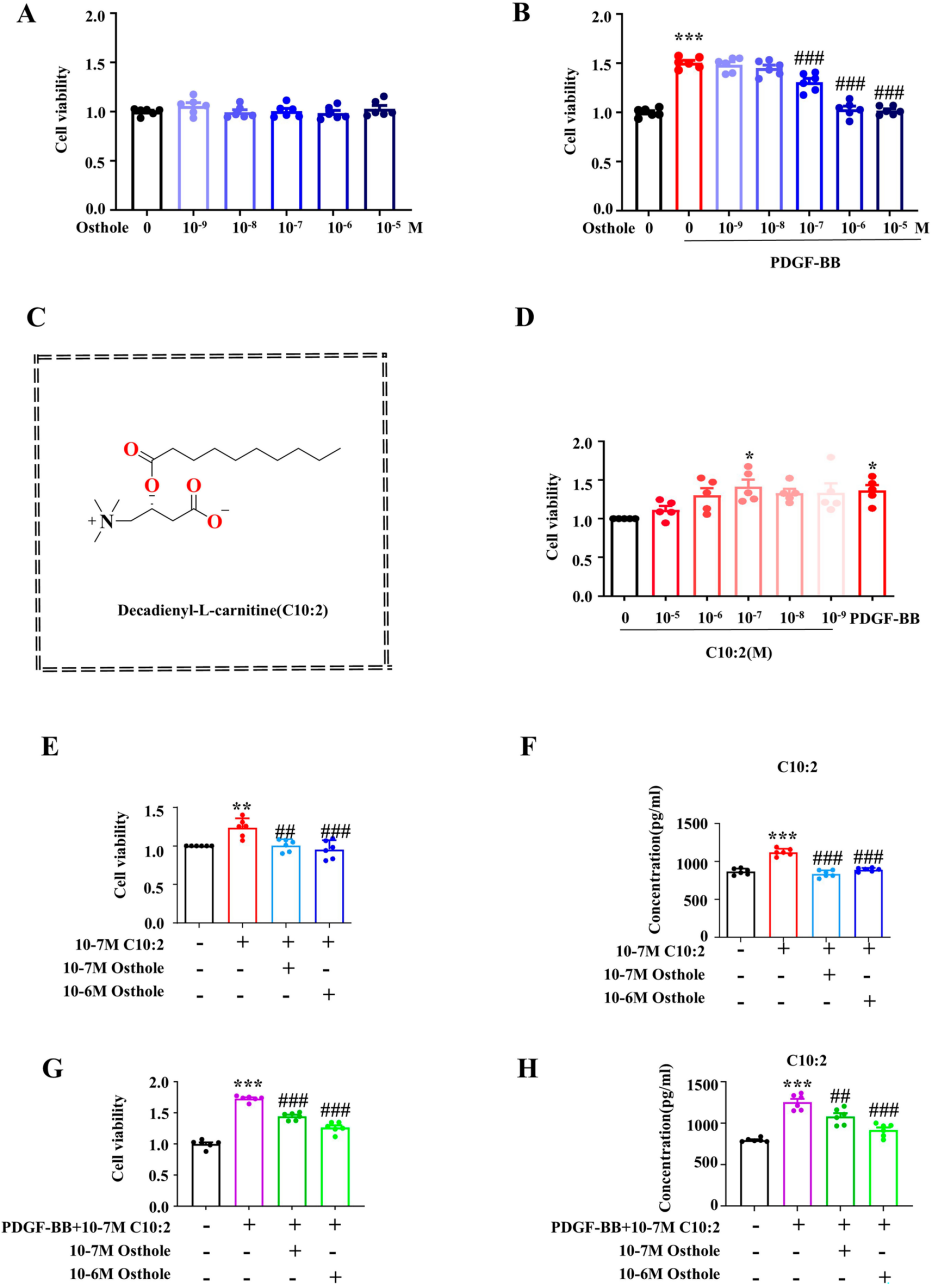
C10:2 promotes PASMC proliferation, that is reversed by osthole. **A**, **B** Effect of osthole on the viability of PASMCs and PDGF-BB–induced PASMCs (n = 6). **C** Chemical structure of C10:2. **D** Cell viability of PASMCs exposed to various concentrations of C10:2 and PDGF-BB (n = 5). **E** Effect of osthole on the viability of PASMCs treated with C10:2 (n = 6). **F** C10:2 concentration was measured by ELISA (n = 6). **G** Effect of osthole on C10:2- and PDGF-BB–mediated proliferation of PASMCs (n = 6). **H** C10:2 concentration was determined by ELISA (n = 6). Data were represented as mean ± SEM. *p < 0.05, **p < 0.01, ***p < 0.001, compared with Control group; ^*#*^*p* < 0.05, ^*##*^*p* < 0.01, ^*###*^*p* < 0.001, compared with PDGF-BB or PDGF-BB + C10:2 group

To investigate the functional metabolite C10:2 in PASMCs proliferation phenotype in PH, the classic in vitro model of PH induced with PDGF-BB was employed [21], then cell viability and C10:2 concentration were examined in PASMCs exposed to PDGF-BB for 24 h, 48 h and 72 h, respectively. We firstly screened the effective dose of osthole agaisnt PASMCs proliferation. As shown in Fig. 2A and B, osthole is not cytoxic to PASMCs at 10^–5^ M to 10^–9^ M, and 10^–6^ M and 10^–7^ M osthole have capability to inhibit PASMCs proliferation induced with PDGF-BB, and were chosen for subsequent experiments. The structure of C10:2 is shown in Fig. 2C. Notably, 10^−7^ M C10:2 has same function as PDGF-BB in promoting PASMCs proliferation. Moreover, exogenous administration of C10:2 could increase the concentration of C10:2 in PASMCs, while osthole obviously suppressed C10:2 concentration as well as C10:2- induced PASMCs proliferation in absence of PDGF-BB (Fig. 2D-F). Simultanously, exogenous administration of C10:2 significantly enhanced cell viability and C10:2 level in PDGF-BB induced PASMCs (Fig. 2G-H). These findings suggested C10:2 could directly promote PASMCs proliferation to promote PVR development, and reversed by osthole treatment.

### Osthole retarded C10:2-mediated cell pyroptosis in vitro

**Fig. 3:**
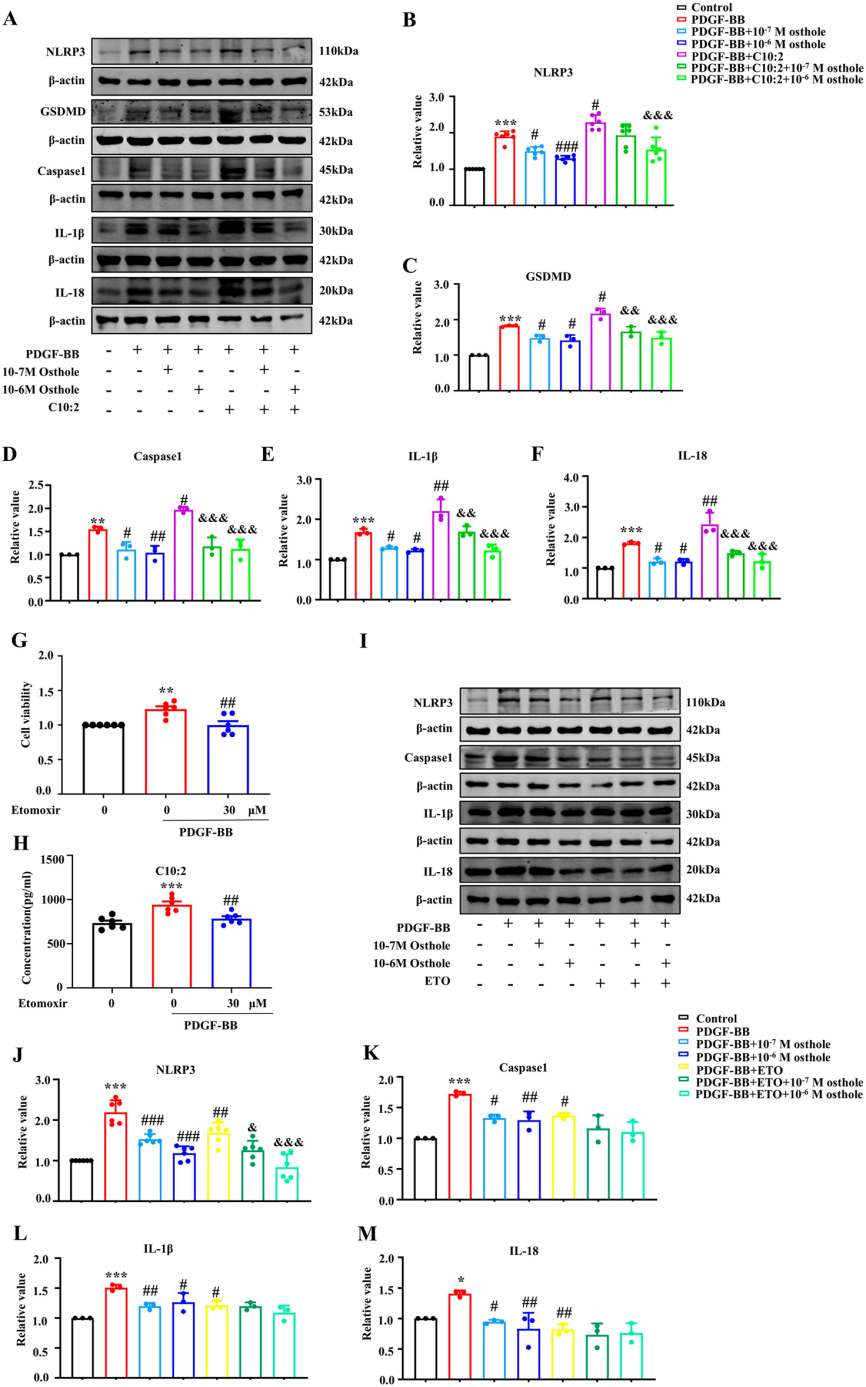
C10:2 induced pyroptosis in PASMCs, which was suppressed by osthole. **A**–**F** The expression levels of NLRP3 (n = 6), GSDMD (n = 3), caspase-1 (n = 3), IL-1β (n = 3), and IL-18 (n = 3) in PASMCs treated with C10:2 were assessed by Western blot analysis. **G** Viability of PASMCs incubated with etomoxir for 24 h was evaluated by MTT assay (n = 6). **H** The concentration of C10:2 was measured by ELISA (n = 6). **I**–**M** Expression levels of NLRP3 (n = 6), caspase-1 (n = 3), IL-1β (n = 3), and IL-18 (n = 3) in PASMCs exposed to etomoxir were determined by Western blot assay. Data were represented as mean ± SEM. *p < 0.05, **p < 0.01, ***p < 0.001, compared with Control group; ^*#*^*p* < 0.05, ^*##*^*p* < 0.01, ^*###*^*p* < 0.001, compared with PDGF-BB group; ^&^*p* < 0.05, ^&&^*p* < 0.01, ^&&&^*p* < 0.001, compared with PDGF-BB + C10:2/ETO group

To further validate the effect of osthole on cell pyroptosis by reducing C10:2 in vitro, exogenous C10:2 and etomoxir were employed to intervene the biosynthesis of C10:2. As shown in Fig. 3, the pyroptosis makers NLRP3, GSDMD, caspase1, IL-18 and IL-1β were significantly increased in model group. In addition, administration of C10:2 further stimulated these proteins upregulated, suggested that C10:2 could accelerate PASMCs pyroptosis, while those changes were reversed by osthole treatment in PASMCs.

Furthermore, C10:2 inhibitor etomoxir was secondly used to investigate the function of C10:2 in cell pyroptosis. Etomoxir, as an inhibitor of CPT1, is a rate-limiting enzyme of C10:2 biosynthesis [13]. As indicated in Fig. 3 G and H**,** 30 μM etomoxir markedly reduced C10:2 concentration to normal level in proliferated PASMCs, therefore, 30 μM etomoxir was used for inhibiting C10:2 synthesis in the following study. As shown in Fig. 3 I-M, inhibition of C10:2 with etomoxir significantly downregulated the expression of pyroptosis proteins NLRP3, caspase1, IL-18 and IL-1β, while these pyroptosis makers were restored by osthole, and there is no difference between the effect of osthole and etomoxir on C10:2 concentration. Together, these findings indicated that C10:2 accelerated PASMCs pyroptosis and reversed by osthole, importantly, osthole is an potential natural inhibitor of C10:2.

### C10:2 facilitated pulmonary vascular remodeling and reversed by osthole

**Fig. 4:**
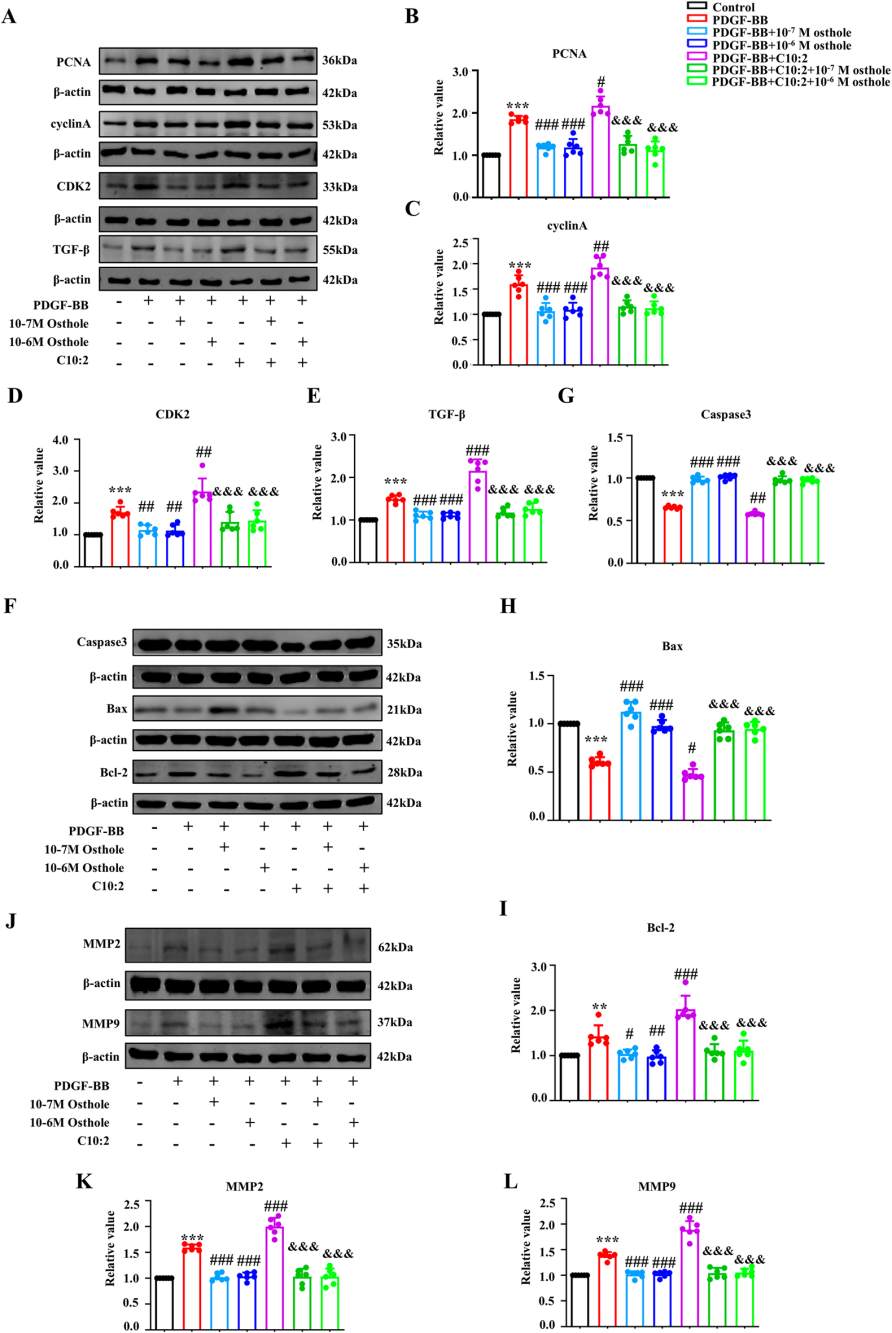
Osthole inhibits C10:2-mediated effects, blocking proliferation, apoptosis resistance, and extracellular matrix remodeling in PASMCs treated with PDGF-BB. **A**–**L** The expression levels of PCNA, cyclin A, CDK2, Caspase-3, Bax, Bcl-2, MMP2, MMP9, and TGF-β in PASMCs were analyzed by Western blot assay (n = 6). Data were represented as mean ± SEM. *p < 0.05, **p < 0.01, ***p < 0.001, compared with Control group;^#^*p* < 0.05,^##^*p* < 0.01, ^*###*^*p* < 0.001, compared with PDGF-BB group; ^&&&^*p* < 0.001, compared with PDGF-BB + C10:2 group

To further address whether C10:2 promoted other phenotypes in PVR, PVR related proteins were examined with Western blot in vitro. As shown in Fig. 4, administration of C10:2 markedly upregulated PCNA, cyclinA, CDK2, Bcl-2, MMP2, MMP9, TGF-β, while significantly dowregulated Caspase3 and Bax in PASMCs compared with PDGF-BB group, indicating that C10:2 may promote PASMCs proliferation, apoptosis inhibition and extracellular matrix remodeling in vitro. More importantly, osthole treatment could restrain PASMCs proliferation by reducing PCNA, cyclinA, CDK2, promote PASMCs apoptosis by modulating Caspase3, Bax and Bcl-2, alleviate extracellular matrix remodeling by downregulating MMP2, MMP9, TGF-β in PH cell model. These results suggested that C10:2 synergistically modulated multiple cell phenotypes to accelerate PVR in vitro and restored by osthole treatment.

### Osthole improved C10:2-induced AMPKα−1 activation and ROS accumulation to ameliorate mitochondrial dysfunction

**Fig. 5:**
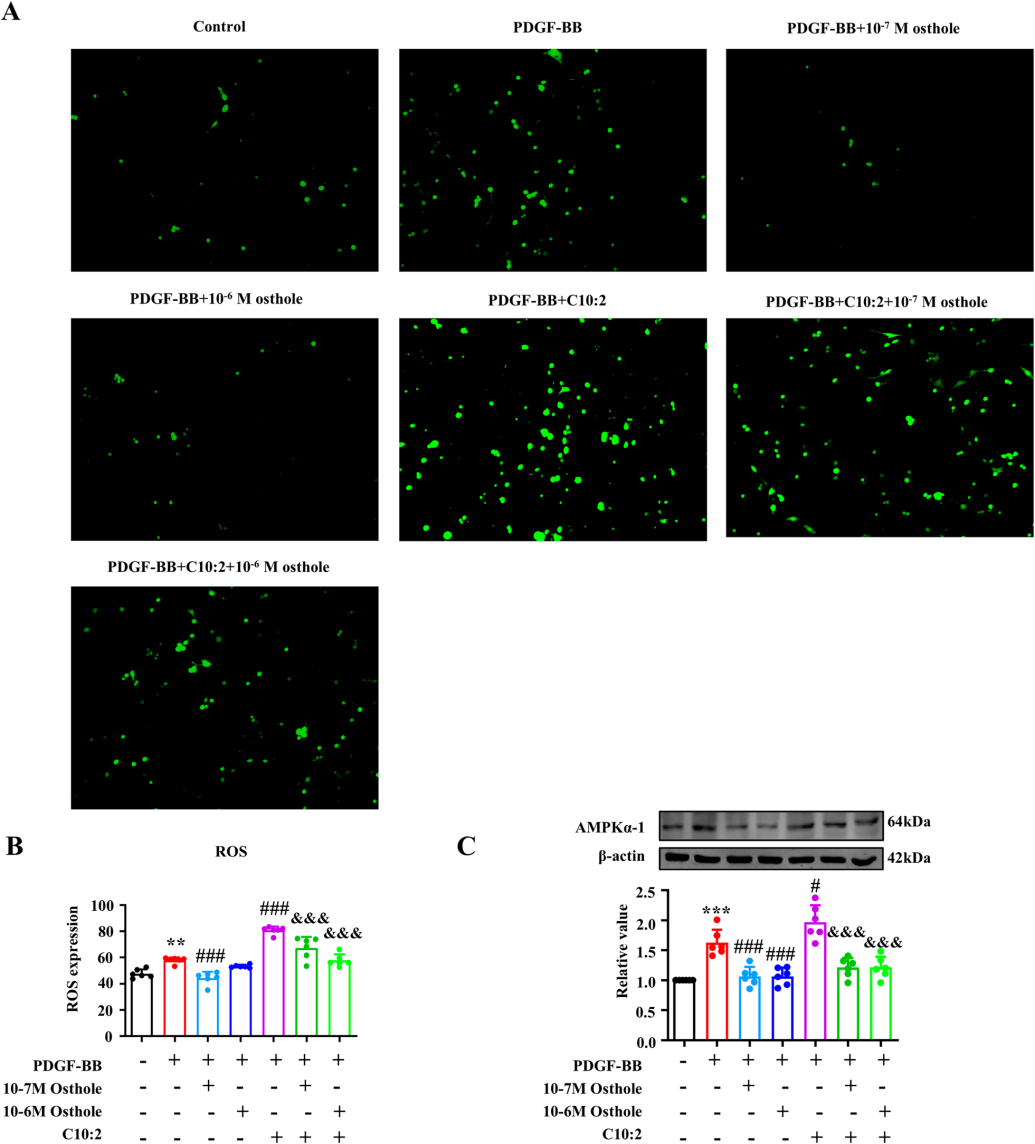
C10:2 induced AMPKα−1 expression and excessive ROS production in PASMCs, that were reversed by osthole treatment. **A**–**B** ROS levels in PASMCs were assessed by immunofluorescence (200 ×, n = 6). **C** AMPKα−1 expression in PASMCs was evaluated by Western blot assay (n = 6). Data were represented as mean ± SEM.**p < 0.01, ***p < 0.001, compared with Control group; ^*#*^*p* < 0.05, ^*###*^*p* < 0.001, compared with PDGF-BB group; ^&&&^*p* < 0.001, compared with PDGF-BB + C10:2 group

PASMCs overproliferation requires a large amount energy to sustain the cellular survival [22]. AMP-dependent protein kinaseα−1 (AMPKα−1), as a energy sensor, is a trigger for PVR [23]. Reactive oxygen species (ROS) overaccumulation serves as a driver in the pathogenesis progression of promoting vascular remodeling [24, 25]. Our results showed that C10:2 significantly induced ROS accumulation and AMPKα−1 activation, while osthole and etomoxir reversed these overexpression (Fig. 5), indicating C10:2 could alert energy crisis and stimulate oxidative stress lead to mitochondrial dysfunction, simutanously, osthole could protect mitochondrial function by reducing C10:2.

### Osthole modulated C10:2/HSP47/NLRP3 axis to alleviate pulmonary vascular remodeling

**Fig. 6:**
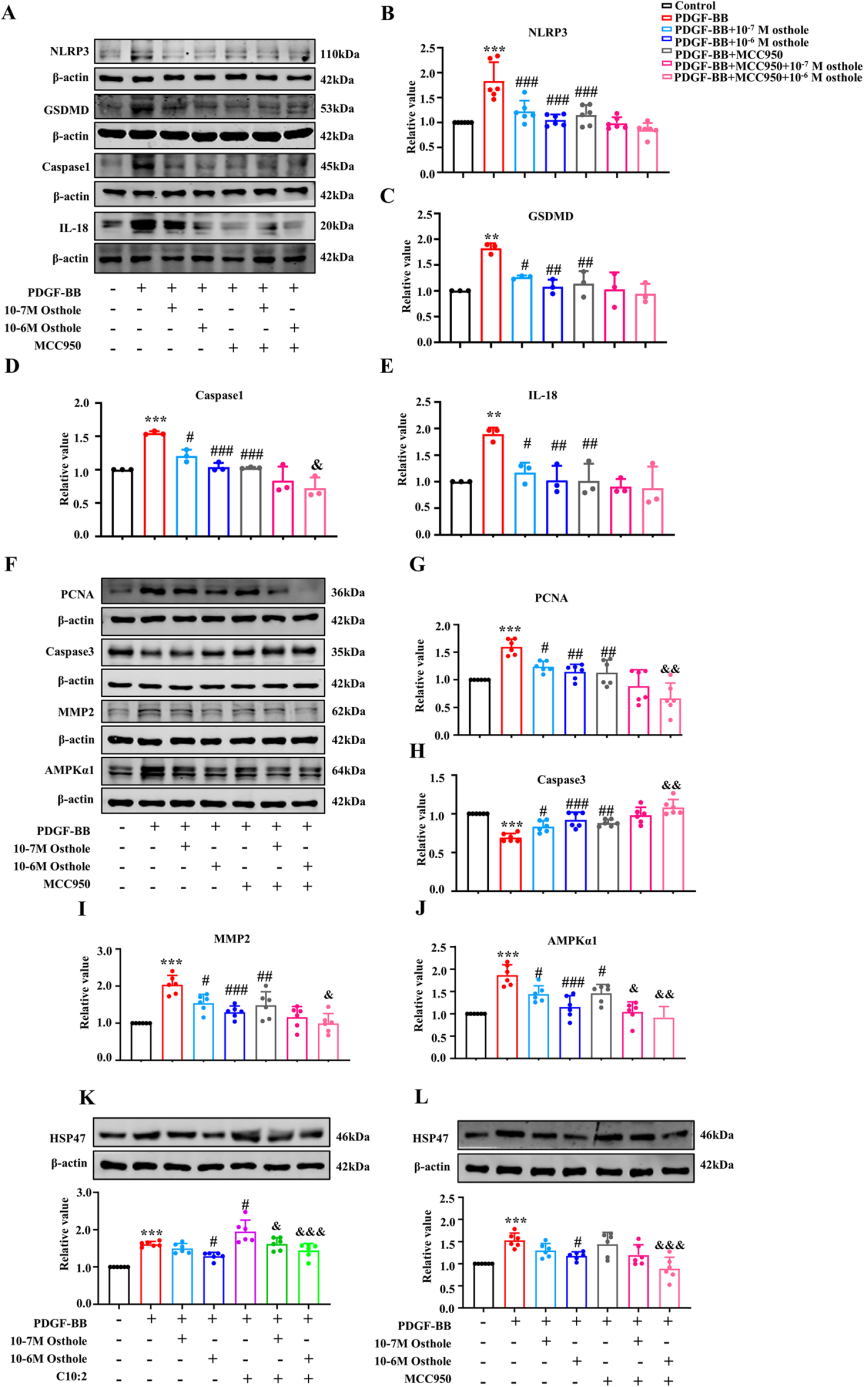
Osthole inhibits the C10:2/HSP47/NLRP3 axis to reverse pulmonary vascular remodeling in vitro. **A**–**J**, **L** Expression levels of NLRP3, GSDMD, Caspase-1, IL-18, PCNA, Caspase-3, MMP2, AMPKα−1, and HSP47 in PASMCs treated with or without the NLRP3 inhibitor MCC950 (n = 3 or 6). **K** HSP47 expression in PASMCs treated with C10:2 (n = 6). Data were represented as mean ± SEM. *p < 0.05, **p < 0.01, ***p < 0.001, compared with Control group; ^*#*^*p* < 0.05,^*##*^*p* < 0.01,^*###*^*p* < 0.001, compared with PDGF-BB group;^&^*p*<0.05, ^&&^*p*<0.01, ^*&&&*^*p* < 0.001 compared with PDGF-BB + MCC950/C10:2 group

To further investigate the downstream signaling pathway of C10:2 facilitating pulmonary vascular remodeling, the crucial pyroptosis marker NLRP3 inflammasome inhibitor MCC950 was employed. As shown in Fig. 6, our data showed that MCC950 was significantly blocked PASMCs pyroptosis by inhibiting NLRP3, GSDMD, Caspase 1, IL-18, and restrained vascular remodeling by affecting PCNA, Caspase3, MMP2, AMPKα−1, while thromboinflammation factor heat shock protein 47 (HSP47) expression was not affected, indicating that HSP47 is the upstream of NLRP3 inflammasome, and C10:2 could modulate pulmonary vascular remodeling via C10:2/HSP47/NLRP3 axis. These changes were all reversed by osthole treatment.

## Discussion

PH is still lacking of specific biomarkers to assist in its early diagnosis and treatment [26]. Here, our findings demonstrated that C10:2 may be served as a potential biomarkerof PVR, and delineated the function of C10:2 in accelerating PVR including facilitating proliferation, apoptosis resistance, extracellular matrix remodeling and mitochondrial dysfunction in PH. Mechanistically, osthole greatly retarded cell pyroptosis to alleviate PVR by the inhibition of C10:2/HSP47/NLRP3 axis in vivo and vitro.

A useful disease biomarker is typically characterized by high sensitivity and specificity, ease of collection and measurement, broad applicability, non-invasiveness or minimal invasiveness, and its ability to indicate key pathological features of the disease [26, 27]. Notably, we found that serum levels of C10:2 accurately reflect the development of core pathological features in PH rats. Thickening of the pulmonary vascular medial layer is primarily driven by excessive proliferation of PASMCs [1, 28]. Importantly, C10:2 exhibited a similar capacity as PDGF-BB to directly promote PASMC proliferation. Moreover, C10:2 is easy to collect, highly sensitive, and convenient to assay. Taken together, these findings highlight C10:2 as an emerging and promising diagnostic biomarker for PVR.

Regarding the phenotype and mechanism of PVR is complex [29], multiple factors in the vascular wall both drive PVR progression [30]. Here, we detected the key markers of PVR in PASMCs by modifying intracellular biosynthesis of C10:2 with etomoxir and exogenous C10:2. Our data demonstrated that C10:2 promoted the expression of PCNA, cyclinA and CDK2, indicating enhanced the combination of cyclinA and CDK2 to form the S-phase landmark complex cyclinA-CDK2 to control cell cycle and stimulate DNA synthesis in PASMCs [31, 32].

Additionally, C10:2 up-regulated anti-apoptotic protein Bcl-2 and down-regulated pro-apoptotic protein Bax to block cytochrome C release, further inhibited the expression of terminal shearing enzyme Caspase-3, finally leading to hinder PASMCs apoptosis [33]. Except for excessive proliferation and apoptosis resistance of PASMCs, extracellular matrix remodeling is additional contributor to PVR [34]. Matrix metalloproteinases (MMP) manipulate extracellular matrix remodeling and PASMCs proliferation [35–37]. MMP2 and MMP9 promote extracellular matrix degradation, thereby decrease vessel compliance, resulting in arterial stiffness [38]. TGF-β promotes cell proliferation resulting in PVR [39, 40]. In this study, we demonstrated C10:2 induces the upregulation of MMP2, MMP9 and TGF-β, thus to promote cell proliferation, decompose extracellular matrix, stimulate extracellular matrix remodeling and vascular stiffness [41].

Cell proliferation needs more oxygen and energy supply. AMPK is activated by energy crisis [34]. A recent study reported that activation of AMPKα1 promoted PASMCs proliferation [23]. Reactive oxygen species (ROS) generation appears to be a necessary step under oxygen deficiency [25]. In addition, acylcarnitine accumulation is sufficient to mediate mitochondrial damage and stimulate ROS overproduction [42, 43]. Consist with this, we observed that C10:2 enhances AMPKα1 activation and induces ROS overproduction response to oxygen and energy stress to promote PASMC survival. Therefore, our findings suggest C10:2 could stimulate pulmonary vascular medial layer thickening and vascular stiffening by directly enhancing PASMCs proliferation and extracellular matrix remodeling, inhibiting PASMCs apoptosis, as well as inducing mitochondrial dysfunction. However, the downstream signal pathway of C10:2 in PVR is still a mystery.

Pyroptosis is recognized as a driver of PVR [44]. C10:2 induced pyroptosis to promote PVR [18]. NLRP3 inflammasome is a supermolecular complex, assembled at mitochondria, activated caspase-1 and GSDMD to induce proinflammatory cytokines release, drivers pyroptosis to induce perivascular inflammation in PH [45–47]. A recent study reported HSP47 contributes to cardiac dysfunction in an NLRP3-dependent manner [48], and regulates thromboinflammation [49–52]. We now have found that C10:2 activated HSP47, NLRP3, caspase-1, GSDMD, to promote inflammatory cytokines release of IL-1β and IL-18, leading to pyroptosis, thus to accelerate PVR. We further demonstrated NLRP3 inhibitor could block C10:2 medicated PVR. Altogether, the metabolic change of C10:2 in PASMCs can drive structural and functional changes of pulmonary vascular by HSP47/NLRP3 axis thus manipulating the PH process.

**Fig. 7:**
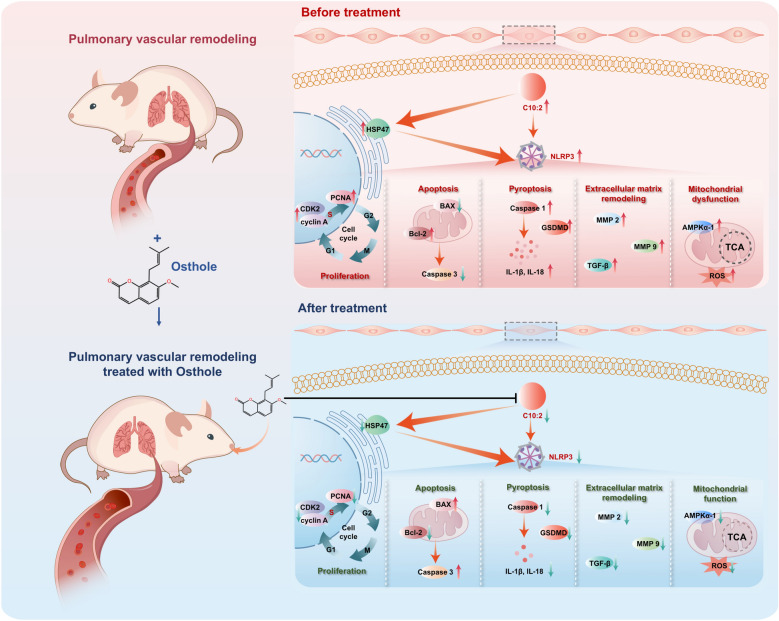
Schematic representation of osthole significantly inhibited pyroptosis to alleviate pulmonary vascular remodeling via C10:2/HSP47/NLRP3 axis

Based on above functional mechanisms of C10:2 in PVR, we found osthole significantly controlled cell cycle and blocked DNA synthesis to inhibit PASMCs proliferation by blocking PCNA, cyclinA and CDK2 in PASMCs; promoted PASMCs apoptosis via modulating Caspase3, Bax and Bcl-2; manipulated extracellular matrix remodeling thereby increased vessel compliance to alleviate arterial stiffness through suppressing MMP2, MMP9 and TGF-β, inactivated energy sensor AMPKα−1 to block energy supply, relived ROS overeaccumulation mediated mitochondrial dysfunction, restrained cell pyroptosis to alleviate vascular inflammation in PVR by the inhibition of HSP47, NLRP3, caspase 1, IL-1β and IL-18. Altogether osthole is a natural inhibitor of C10:2, ameliorates PASMCs pyroptosis to alleviate PVR via C10:2/HSP47/NLRP3 axis (Fig. 7).

## Conclusion

This study is the first to identify C10:2 as a potential biomarker for PVR in PH and to elucidate the novel biological mechanism by which C10:2 facilitates multiple cellular phenotypes to promote vascular remodeling. Importantly, our findings reveal a new therapeutic mechanism in which osthole inhibits pyroptosis to ameliorate PVR via the C10:2/HSP47/NLRP3 axis. Collectively, our results demonstrate how the small molecular metabolite C10:2 modulates cellular signaling to drive the pathological progression of PH. These insights provide not only an invaluable diagnostic biomarker but also a promising candidate drug for PH treatment.

## Supplementary Information

Additional file 1

## Data Availability

The datasets supporting this study’s conclusions are accessible through the corresponding author upon a reasonable request.

## Abbreviations

AMPK: AMP-dependent protein kinase
Bcl-2: B-cell lymphoma-2
Bax: Bcl2-Associated X
CDK2: Cyclin-dependent kinases 2
C10:2: Decadienyl-l-carnitine
ETO: Etomoxir
HSP47: Heat shock protein 47
IL-1β: Interleukin-1β
IL-18: Interleukin-18
MCT: Monocrotaline
MMP2: Matrix metallopeptidase 2
MMP9: Matrix metallopeptidase 9
NLRP3: NOD-,LRR-and pyrin domain-containing protein 3
PASMCs: Pulmonary artery smooth muscle cells
PCNA: Proliferating cell nuclear antigen
PDGF-BB: Platelet-derived growth factor-BB
PH: Pulmonary hypertension
PVR: Pulmonary vascular remodeling
ROS: Reactive oxygen species
TGF-β: Transforming growth factor β
