# Supplementary material for: A novel role for decadienyl-L-carnitine in pulmonary vascular remodeling and the underlying interventional mechanism of osthole
Source: Chin Med. 2026 Mar 16;21:87. doi: 10.1186/s13020-026-01362-8 (PMC12990433; doi:10.1186/s13020-026-01362-8)
Supplement: Supplementary file 1 — Additional file 1 [file 13020_2026_1362_MOESM1_ESM.docx]

**Supplementary material**

**Figure S1-S6**


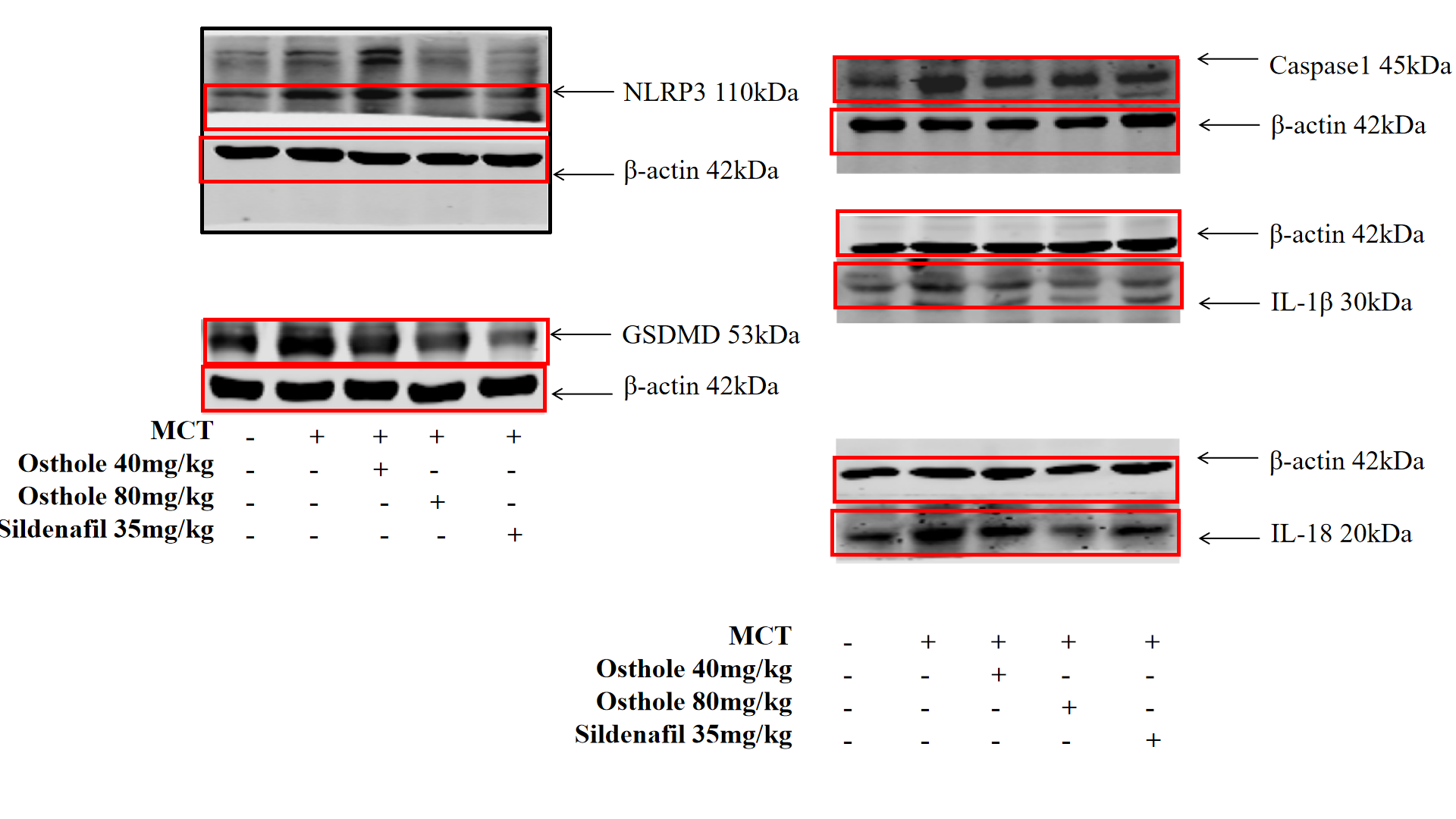


Figure S1. The original bands of osthole inhibited pyroptosis in lung tissue.


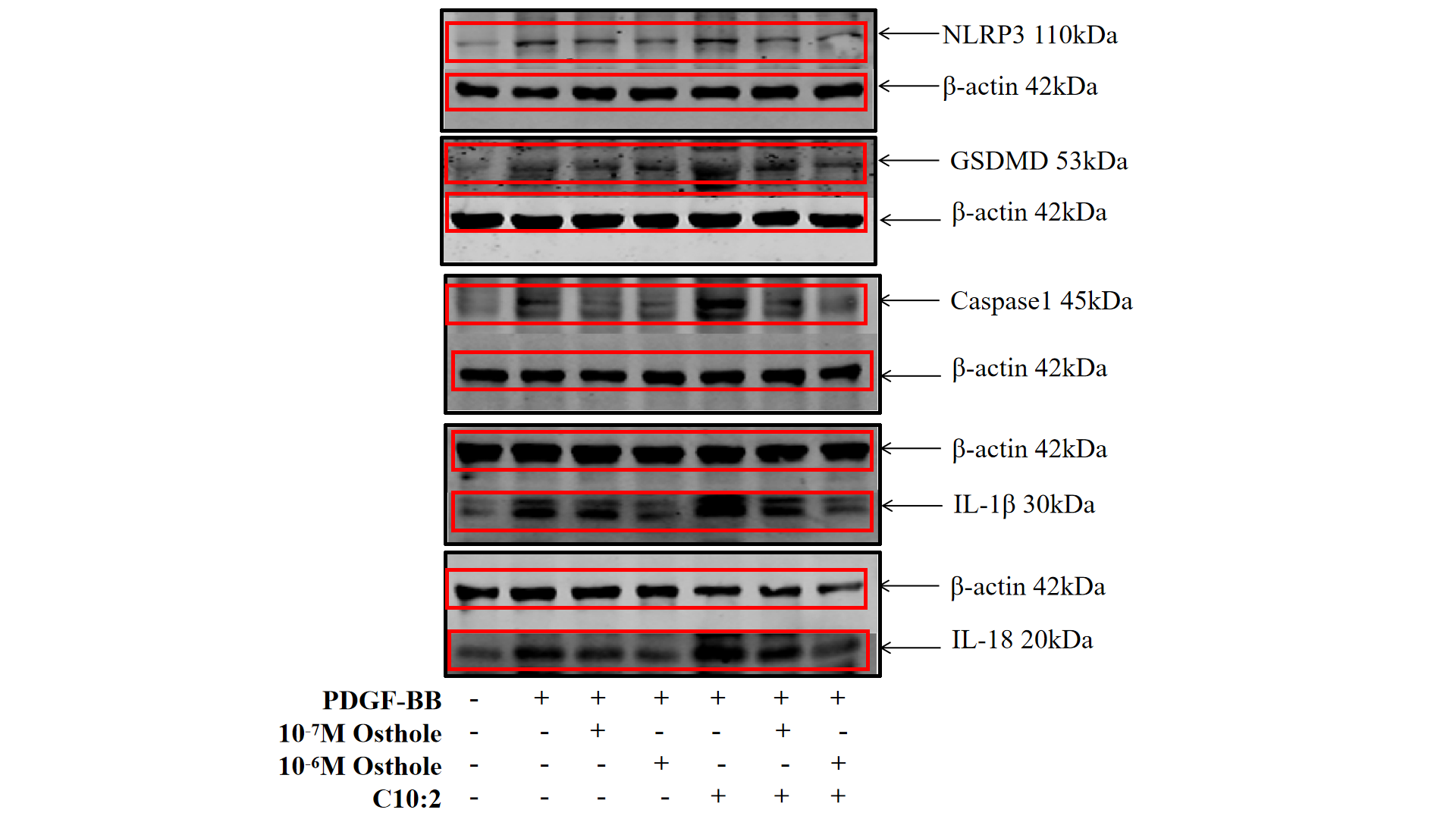


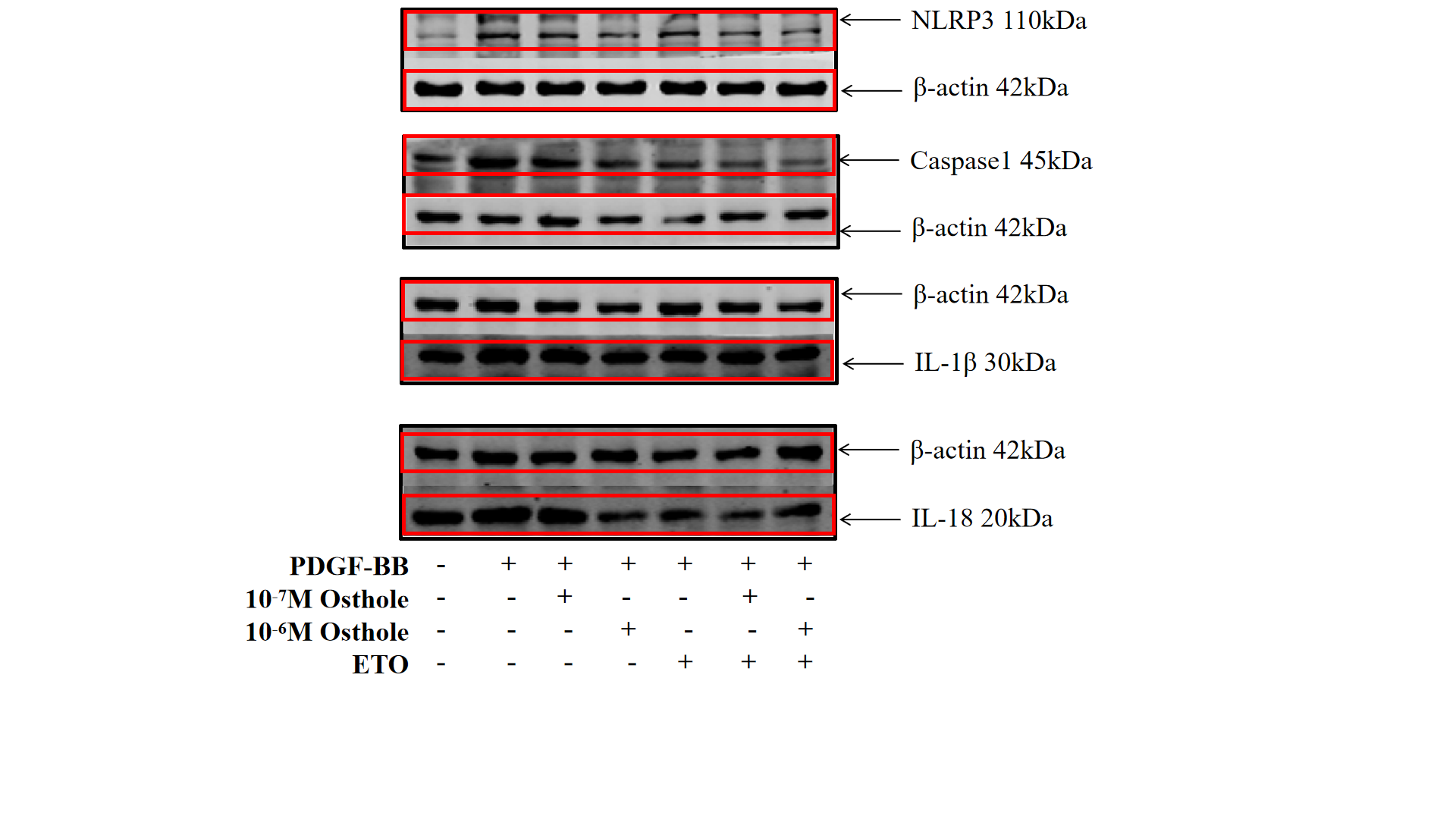


Figure S2. The original bands of C10:2 promoted ferroptosis and ETO inhibited pyroptosis in PASMCs.


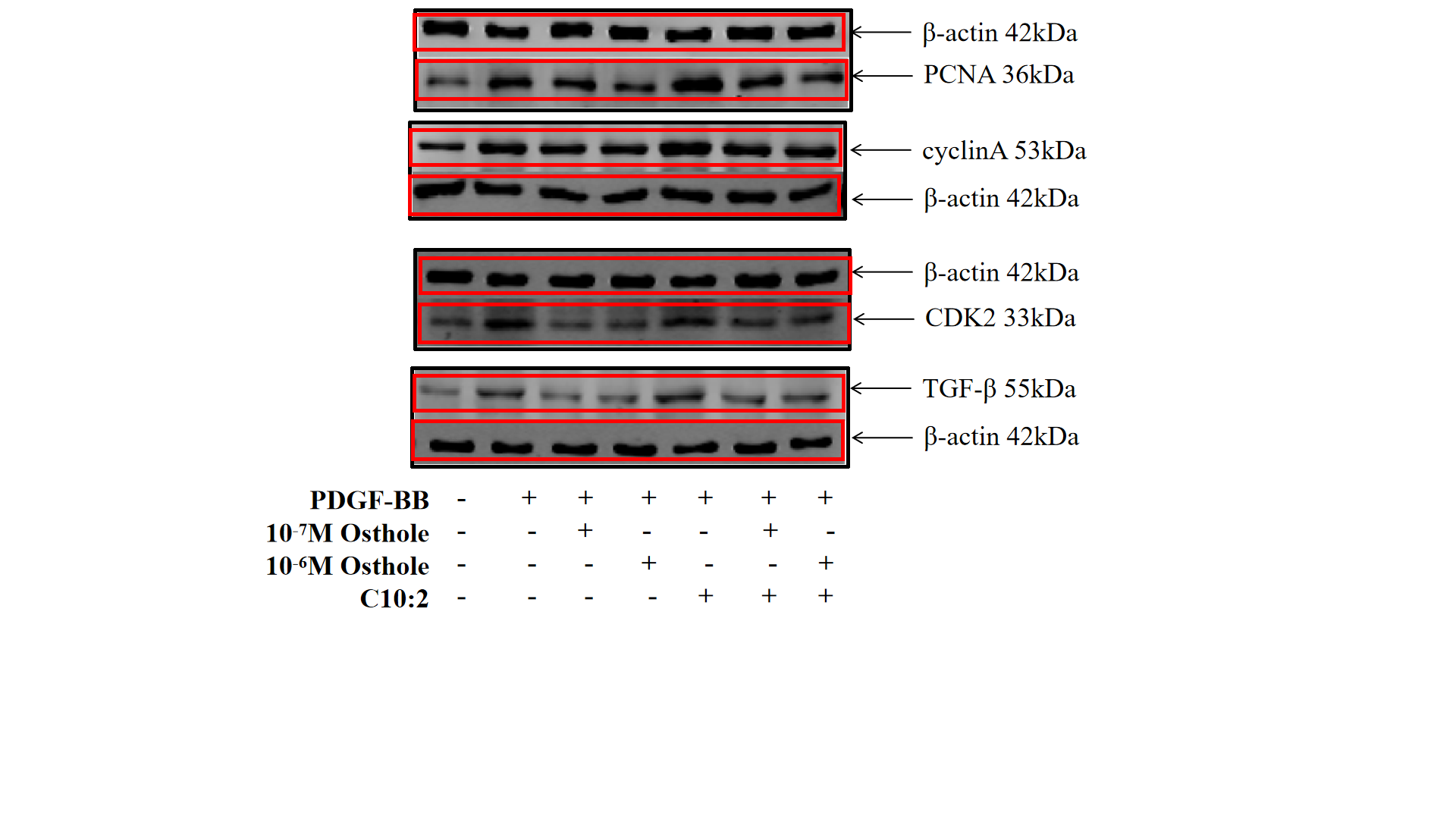


Figure S3. The original bands of C10:2 promoted cell proliferation in PASMCs.


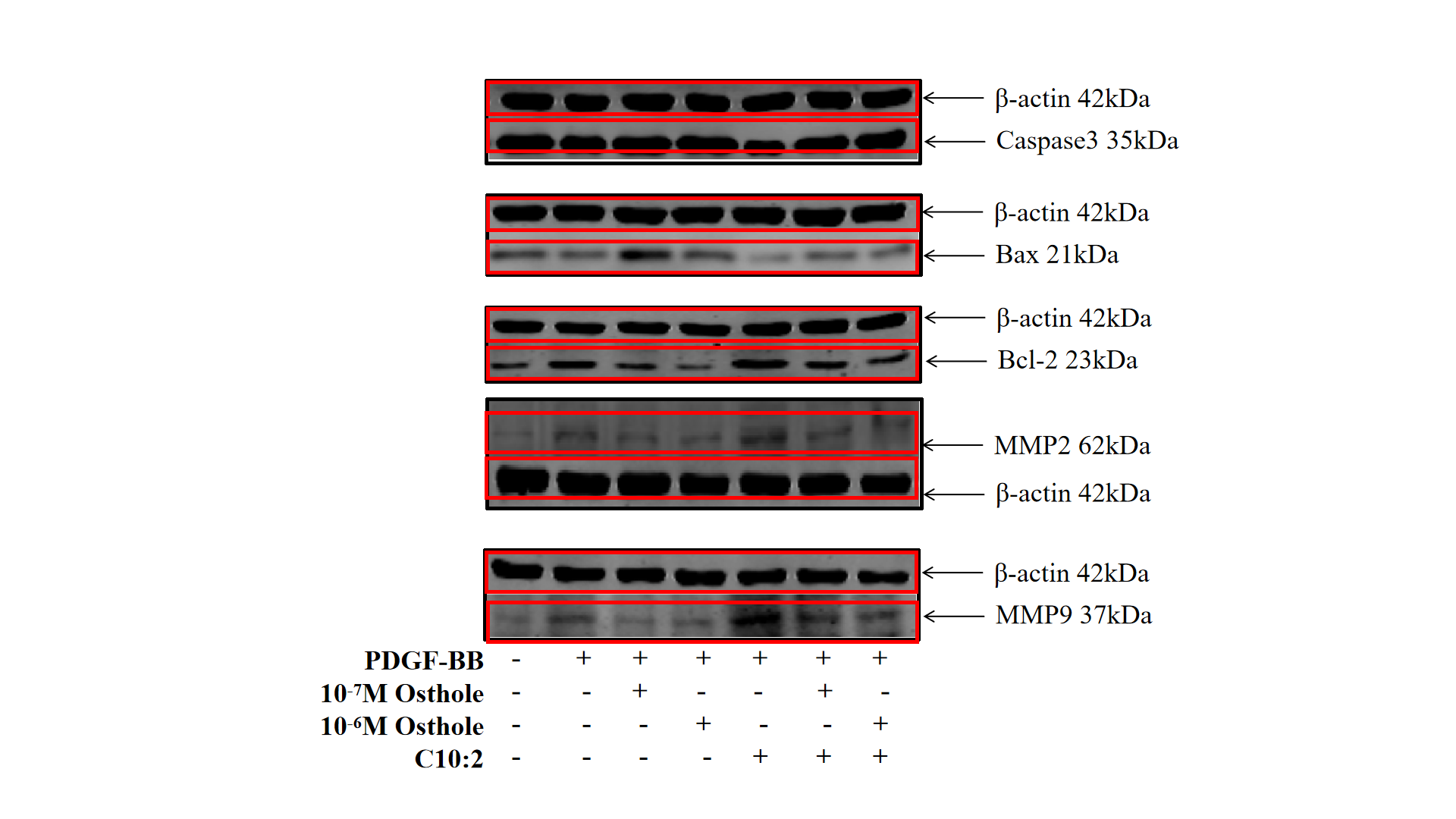


Figure S4. The original bands of C10:2 inhibited cell apoptosis and triggered extracellular matrix remodeling in PASMCs.


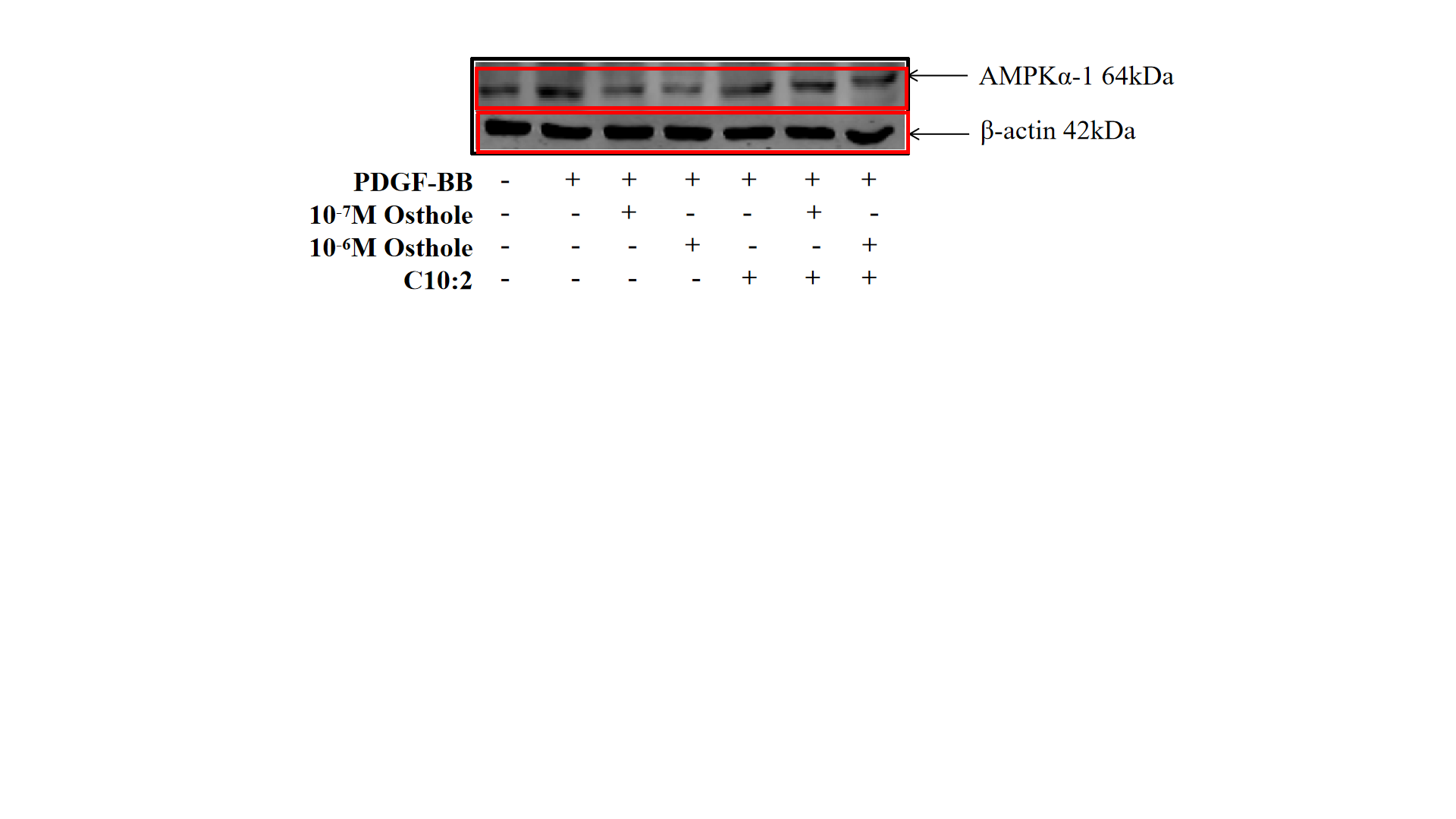


Figure S5. The original bands of osthole inhibited AMPKα-1 activation in PASMCs.


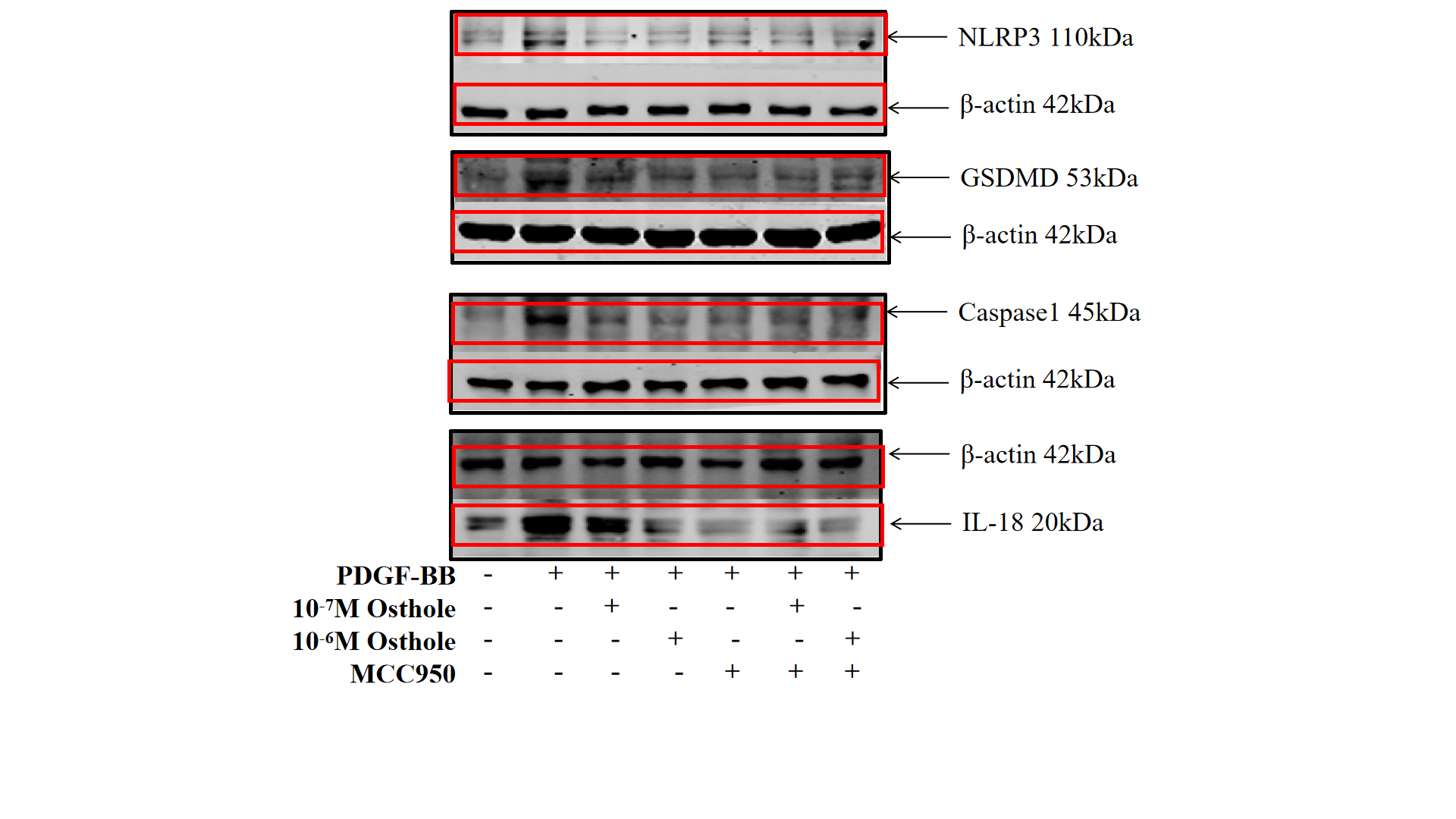


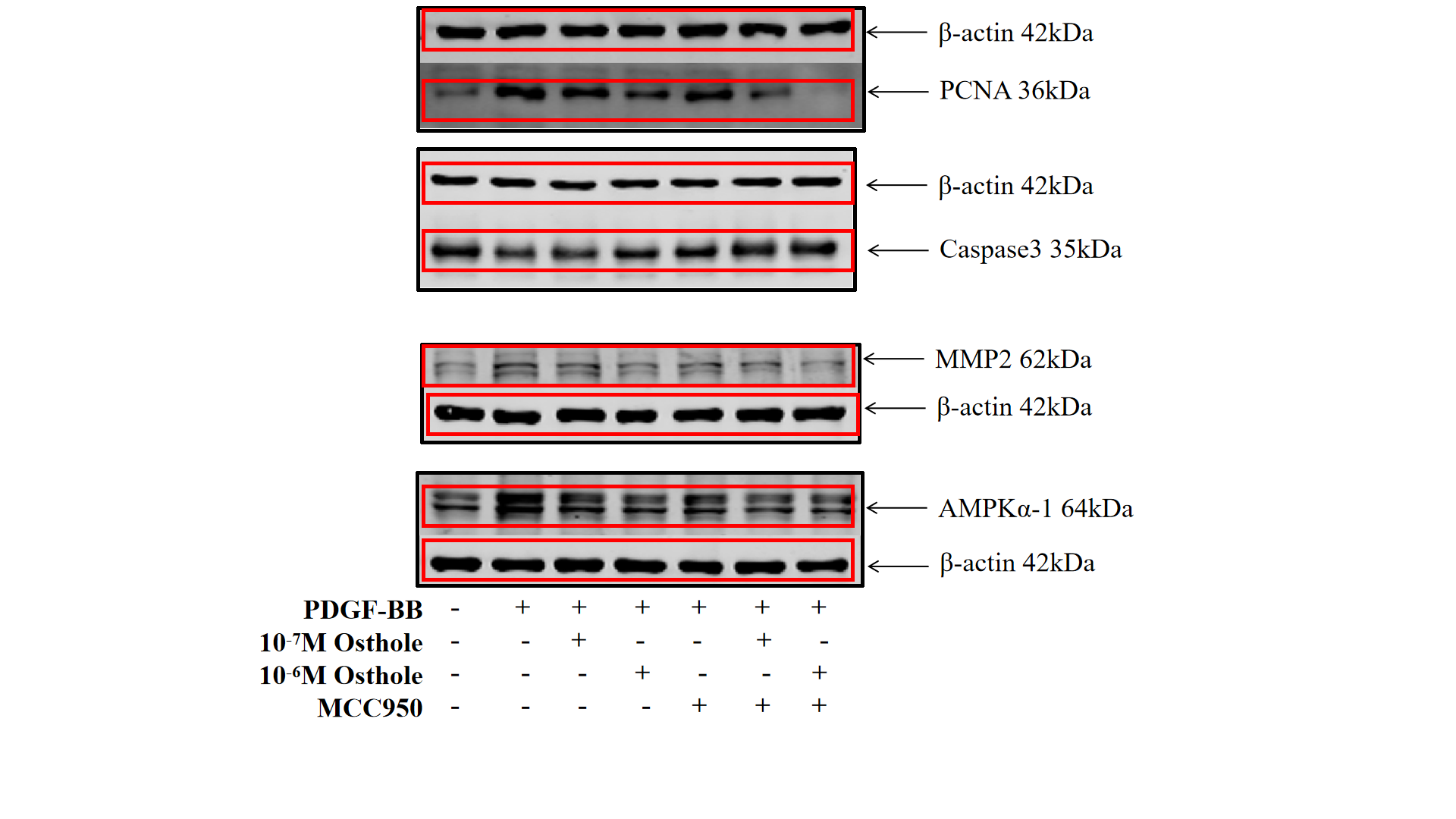


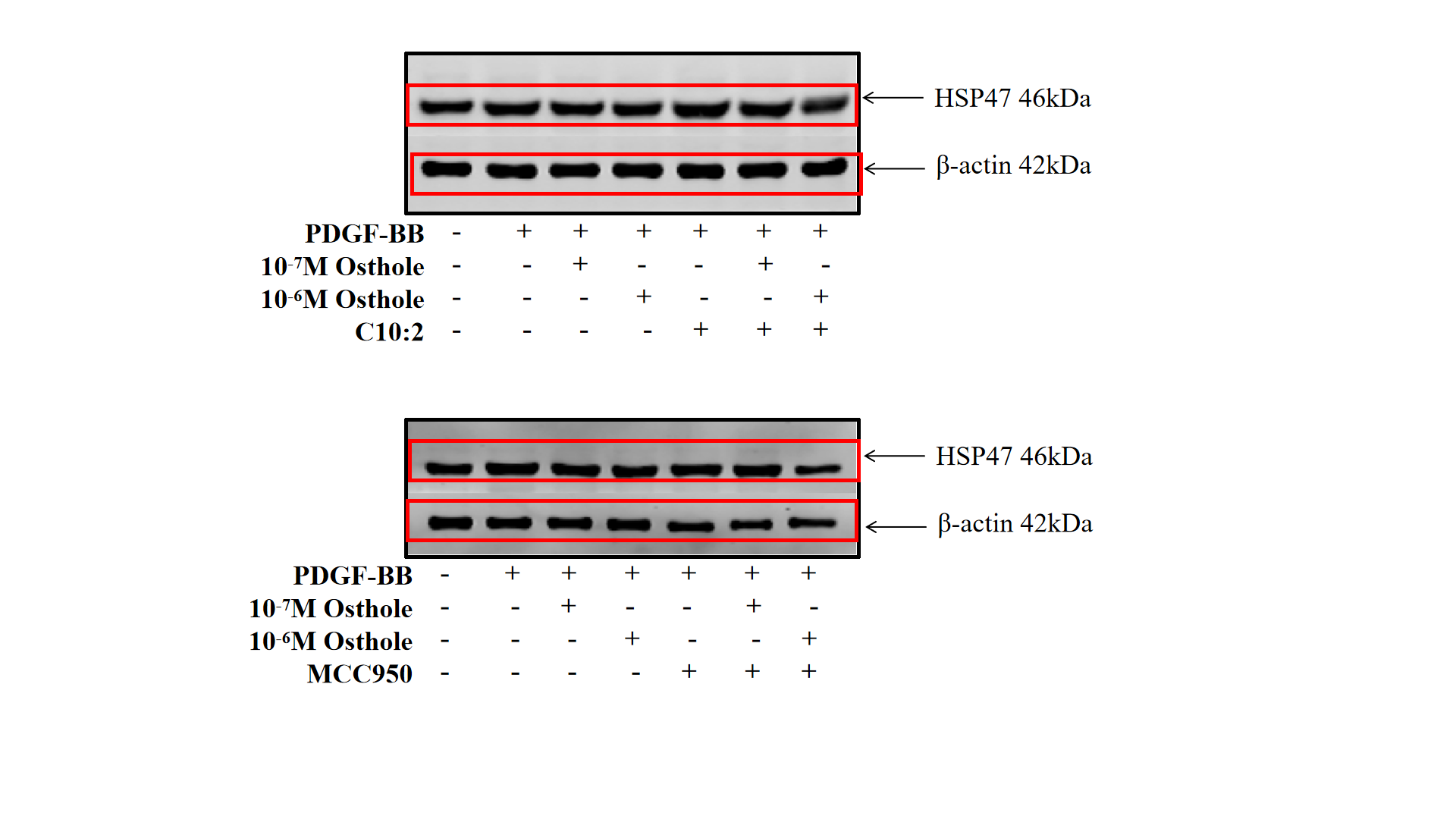


Figure S6. The original bands of the effects of MCC950 on pyroptosis, proliferation, apoptosis, energy metabolism in PASMCs, and the regulatory role of osthole.
